# Arming T Cells with a gp100-Specific TCR and a CSPG4-Specific CAR Using Combined DNA- and RNA-Based Receptor Transfer

**DOI:** 10.3390/cancers11050696

**Published:** 2019-05-20

**Authors:** Bianca Simon, Dennis C. Harrer, Beatrice Schuler-Thurner, Gerold Schuler, Ugur Uslu

**Affiliations:** 1 Department of Dermatology, Friedrich-Alexander-Universität Erlangen-Nürnberg (FAU), Universitätsklinikum Erlangen, 91054 Erlangen, Germany; bianca.simon@uk-erlangen.de (B.S.); dennis.harrer@uk-erlangen.de (D.C.H.); beatrice.schuler-thurner@uk-erlangen.de (B.S.-T.); gerold.schuler@uk-erlangen.de (G.S.); 2 Division of Genetics, Department of Biology, Friedrich-Alexander-Universität Erlangen-Nürnberg (FAU), 91058 Erlangen, Germany

**Keywords:** cancer, melanoma, immune escape, antigen loss, immunotherapy, chimeric antigen receptor, electroporation, lentivirus, lentiviral transduction

## Abstract

Tumor cells can develop immune escape mechanisms to bypass T cell recognition, e.g., antigen loss or downregulation of the antigen presenting machinery, which represents a major challenge in adoptive T cell therapy. To counteract these mechanisms, we transferred not only one, but two receptors into the same T cell to generate T cells expressing two additional receptors (TETARs). We generated these TETARs by lentiviral transduction of a gp100-specific T cell receptor (TCR) and subsequent electroporation of mRNA encoding a second-generation CSPG4-specific chimeric antigen receptor (CAR). Following pilot experiments to optimize the combined DNA- and RNA-based receptor transfer, the functionality of TETARs was compared to T cells either transfected with the TCR only or the CAR only. After transfection, TETARs clearly expressed both introduced receptors on their cell surface. When stimulated with tumor cells expressing either one of the antigens or both, TETARs were able to secrete cytokines and showed cytotoxicity. The confirmation that two antigen-specific receptors can be functionally combined using two different methods to introduce each receptor into the same T cell opens new possibilities and opportunities in cancer immunotherapy. For further evaluation, the use of these TETARs in appropriate animal models will be the next step towards a potential clinical use in cancer patients.

## 1. Introduction

Adoptive transfer of T cells transfected with tumor-specific T cell receptors (TCRs) or chimeric antigen receptors (CARs) has already been successfully used in clinical trials treating patients suffering from several types of solid and hematologic malignancies [1,2,3,4,5]. Especially for therapy-refractory B-cell acute lymphoblastic leukemia (ALL) and diffuse large B-cell lymphoma, CD19-directed CAR T cell therapy revealed impressive response rates in clinical trials [5,6,7,8]. This has resulted in the recent approval of two CD19 CAR T cell constructs in the United States and the European Union [5,6,7,8].

However, a major hurdle in the use of adoptively transferred T cells, especially for the use in solid tumors, is posed by mechanisms of tumor cells that enable them to escape T cell recognition [9,10,11]. Occurrences of, e.g., tumor antigen loss, downregulation of the antigen presenting machinery, or defects in antigen processing are collectively known as “immune escape mechanisms” and give rise to disease progression after an initial response [9,10,11]. Thus, new strategies to improve the adoptive transfer of engineered T cells are required [12,13,14]. A possible approach is the introduction of not only one but two tumor-specific receptors into the same T cell in order to generate T cells expressing not only one, but two additional tumor antigen-specific receptors. This would reduce the risk of immune escape, as it is less likely that both tumor antigens recognized by the same T cell will be lost or downregulated by the same tumor cell at the same time. Additionally, a multi-hit therapy by attacking tumor cells via more than one target antigen simultaneously may result in a more efficient tumor cell killing.

In our previous studies, we have already generated so called “T cells expressing two additional receptors” (TETARs) with two TCRs specific for human immunodeficiency virus (HIV)-epitopes [15] or melanoma antigens [16] using co-electroporation of receptor-encoding mRNAs. These TETARs equivalently responded to both epitopes with regard to cytokine secretion and cytotoxic function [15,16]. However, by equipping the same T cells simultaneously with two TCRs, tumor cells could still escape immune recognition by defects in antigen processing or loss of human leukocyte antigen (HLA) expression. This could be circumvented by, e.g., co-transfection of the T cells with a CAR and a TCR: While TCRs recognize intracellular tumor antigens that are presented on the cell surface by major histocompatibility complex (MHC) molecules [17], CARs bind to unprocessed tumor surface antigens independent of MHC restriction and antigen processing [18,19]. Thus, we generated TETARs by simultaneous transfection of a second-generation CAR and a conventional TCR using again co-electroporation of receptor-encoding mRNAs [20]. We could confirm that a CAR and a TCR can be functionally combined, as these TETARs produced cytokines and were cytotoxic upon recognition of each of their cognate antigens, while no reciprocal inhibition of the receptors occurred [20].

Using only transient RNA-based transfection to introduce the two receptors into the same T cells to generate TETARs represents a safer method, as potential side effects will be transient as well. At the same time, a clinical application would require repetitive infusions and thus, a higher amount of engineered T cells [21,22,23]. The use of only DNA-based receptor transfer to introduce both receptors stably—and not transiently—into the same T cell, however, would most likely result in higher rates of severe side effects [24], complicating its clinical application as well. Thus, the logical strategy would be to stably transfer receptors which have already proven to be effective and safe, and to transiently transfer receptors into the same T cell which are likely potent, but potentially more dangerous regarding on-target off-tumor toxicities. Thus, an effective strategy could be to stably transfer receptors which have already proven to be effective and safe, and to transiently transfer receptors into the same T cells which have been shown to be potent, but potentially more dangerous regarding side effects. In this context, the receptor, which is virally transduced into the T cell using receptor-encoding DNA, should have a strong and permanent anti-tumor effect, whereas the receptor, which is transfected into the same T cell using receptor-encoding RNA, should have an additional “boost” effect at the beginning of the therapy. This initial “boost” effect could massively increase the pressure on the tumor by enhanced induction of direct tumor-cell killing and rapid on-site T cell expansion and could be an effective and fast way to eradicate large quantities of tumor cells.

The CSPG4 antigen (chondroitin sulfate proteoglycan 4, also known as melanoma-associated chondroitin sulfate proteoglycan, MCSP, or high molecular weight melanoma-associated antigen, HMW-MAA) is expressed in 90% of melanoma lesions [25] and other malignancies, e.g., gliomas and sarcomas [26,27]. It is a major driver of melanoma progression by influencing adhesion/spreading, migration, invasion, and metastasis [25]. It is also expressed on healthy tissue, e.g., precursor cells of hair follicle and epidermal cells, as well as on endothelial cells and on activated pericytes [28,29]. The gp100 antigen (also known as premelanosome protein, PMEL) is an intracellular transmembrane glycoprotein enriched in melanosomes, which is involved in the synthesis of melanin [30]. Thus, both antigens, CSPG4 and gp100, may represent effective target antigens for the use in adoptive T cell transfer.

The aim of this study was to simultaneously transfer two receptors into the same T cell to generate TETARs by combining stable DNA- and transient RNA-based receptor transfer. Since the gp100 antigen has been previously used as a target antigen in adoptive T cell therapy and has proven to be an effective as well as a safe target antigen [1,31], we decided to stably introduce a gp100-specific TCR into the T cells via lentiviral transduction. Due to the more distributed expression pattern of CSPG4 in malignant as well as healthy tissue, we additionally introduced a CSPG4-specific second-generation CAR transiently into the same T cell via electroporation of receptor-encoding RNA. Following pilot experiments to optimize the combined DNA- and RNA-based receptor transfer, we examined the functional activity, i.e., cytokine production and cytotoxicity, of TETARs and compared them to T cells either transfected with the TCR only or the CAR alone to provide a proof of principle of this novel approach.

## 2. Results

### 2.1. Generation of TETARs

**Figure 1:**
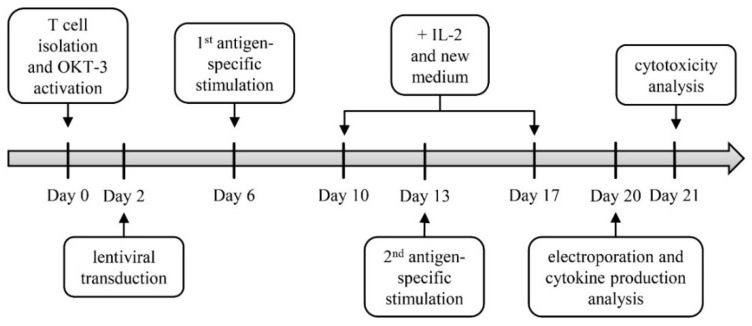
Experimental procedure for the generation of T cells expressing two additional receptors (TETARs). CD8^+^ T cells were first isolated from peripheral blood mononuclear cells (PBMCs) and subsequently activated with OKT-3, CD28 antibody, and IL-2 (T cell isolation and activation). After two days, T cells were lentivirally transduced with the gp100 TCR virus (lentiviral transduction). On day 6 and 13, antigen-specific stimulation of transduced T cells with gp100 peptide-loaded A375M melanoma cells was performed (antigen-specific stimulation). New medium and IL-2 was added to the cells on day 10 and 17 (+ IL-2 and new medium). Transduced T cells were then electroporated with mRNA encoding the CSPG4-specific chimeric antigen receptor (CAR), and functionality assays, i.e., analysis of cytokine secretion and cytotoxicity, were conducted at day of or one day after electroporation. In addition, a receptor expression analysis was performed in a time-course experiment.

At the beginning of experimental procedures, healthy donor T cells were extracted from peripheral blood mononuclear cells (PBMCs) via magnetic-activated cell sorting (MACS) and short-time activated with the anti-CD3 antibody OKT-3, IL-2, and αCD28 (Figure 1). At day two, T cells were lentivirally transduced with a gp100-specific TCR. Non-transduced cells served as a negative control. In order to directly expand transduced cells, antigen-specific stimulation was performed with A375M melanoma cells pulsed with the HLA-A2-restricted peptide gp100_280–288_ for one week (Figure 1). This antigen-specific stimulation of cells was performed twice consecutively, adding IL-2 and fresh medium at day four after each stimulation. At the same time, non-transduced cells were again activated with OKT-3, IL-2, and αCD28. On day 20, half of the transduced cells as well as non-transduced cells were electroporated with a CSPG4-specific CAR leading to the following T cell conditions: non-transduced cells (mock), CSPG4 CAR-transfected cells (CAR T cells; CAR only), gp100 TCR-transduced T cells (TCR T cells; TCR only), and TCR-transduced plus CAR-transfected T cells (TETARs). After T cell engineering, cells were examined for their receptor expression levels. In the next step, functionality of these TETARs, i.e., cytokine production and cytotoxicity, was analyzed and compared to CAR T cells and TCR T cells (Figure 1).

### 2.2. Gp100-Specific TCR T Cells Can Be Efficiently Transfected with a CSPG4-Specific CAR Using mRNA Electroporation

In order to determine whether the lentivirally transduced T cells can be additionally transfected with mRNA coding for a tumor-specific CAR, the surface expression of the gp100-specific TCR and the CSPG4-specific CAR were examined following RNA electroporation. The expression of the gp100-specific TCR was confirmed using an MHC-Dextramer (HLA-A*0201/YLEPGPVTV), whereas successful CAR transfection was detected via an anti-IgG1 antibody. Successive TCR and CAR stainings were performed in a time-course experiment at 4, 8, and 24 h after CAR transfection. 

**Figure 2:**
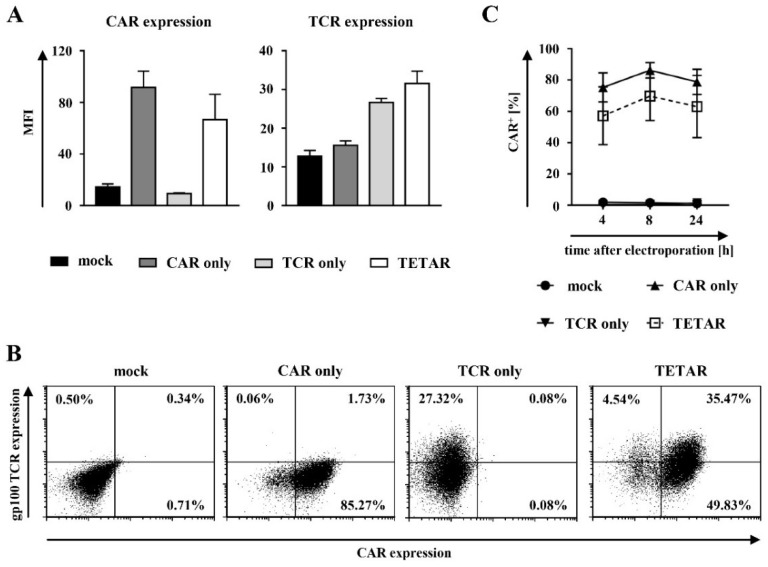
Gp100-specific T cell receptor (TCR) T cells can be additionally equipped with a CSPG4-specific CAR via mRNA electroporation. (**A**–**C**) CD8^+^ T cells were lentivirally transduced with a gp100-specific TCR (TCR only) and electroporated with mRNA coding for the CSPG4-specific CAR (TETARs), as indicated. Non-transduced T cells were either transfected without mRNA (mock) or with CSPG4-specific CAR mRNA (CAR only). Mock-transfected cells served as a negative control. (**A**,**B**) The surface expression of the gp100-specific TCR and the CSPG4-specific CAR at eight hours after electroporation are shown. (**A**) Average geometric mean values of three independent experiments with SEM and (**B**) dot plots of one representative out of three independent experiments are depicted. (**C**) Mean percentages of CAR-positive cells of three independent experiments ± SEM are shown. The *p*-values were calculated by unpaired Student’s *t*-test and are listed in Table S1.

Mock-transfected cells showed no or only few unspecific TCR- or CAR-positive cell populations (Figure 2A,B). The gp100-specific TCR was constantly expressed on lentivirally transduced cells indicated by the similar expression pattern exhibited by TCR T cells and TETARs (Figure 2A and Figure S1). Regarding transient CAR expression, an increase from 4 to 8 h after RNA-electroporation was observed in both CAR T cells and TETARs, with a subsequent decrease of receptor expression at 24 h after transfection (Figure 2A and Figure S1). TETARs revealed expression of both receptors on the cell surface, indicated by a single double-positive population as seen in the dot plots (Figure 2B). Further analysis of transfection efficacy displayed no significant differences in CAR-positive cells when cells engineered with the CAR only were compared to TETARs (Figure 2C and Table S1). 

Taken together, these results demonstrate that it is feasible to generate TETARs through additional mRNA-transfection of already lentivirally transduced T cells. In addition, TETARs showed an equal transfection efficacy in comparison to CAR T cells and TCR T cells.

### 2.3. TETARs Antigen-Specifically Secrete Cytokines

**Figure 3:**
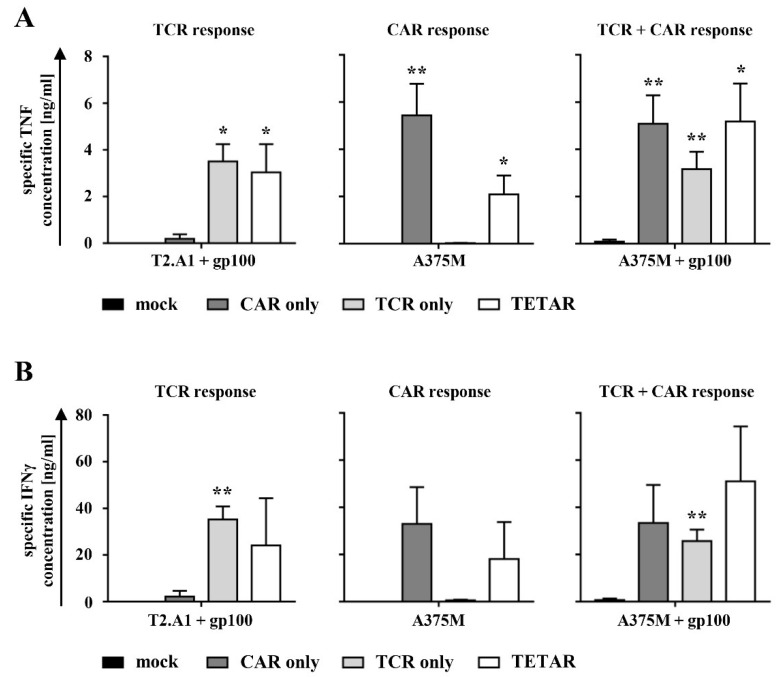
TETARs specifically produced cytokines after antigen encounter. (**A**,**B**) CD8^+^ T cells were lentivirally transduced with a gp100-specific TCR (TCR only) and electroporated with mRNA coding for the CSPG4-specific CAR (TETARs). Non-transduced T cells were either transfected without mRNA (mock) or with CSPG4-specific CAR mRNA (CAR only). Mock-transfected cells were used as a negative control. Following overnight stimulation of T cells with either gp100 peptide-loaded or unpulsed T2.A1 (HLA-A2^+^, CSPG4^−^, gp100^−^) and A375M (HLA-A2^+^, CSPG4^+^, gp100^−^) tumor cells, the production of cytokines was measured in a cytometric bead array (CBA). (**A**) Specific secretion of tumor necrosis factor (TNF) and (**B**) interferon-gamma (IFNγ) were calculated by subtracting the number of produced cytokines after co-incubation with unpulsed T2.A1 target cells, which served as a negative control. (**A**,**B**) Mean values of four independent experiments with SEM are shown. The *p*-values were calculated by unpaired Student’s *t*-test and are listed in Table S2. ** *p* ≤ 0.01, * *p* ≤ 0.05.

In the next step, antigen-specific cytokine production of engineered T cells in response to tumor cells, expressing either one of the antigens or both, was examined. Lentivirally transduced T cells were subsequently electroporated either without mRNA (TCR T cells; TCR only) or with mRNA coding for the CSPG4-specific CAR (TETARs). Non-transduced cells were either transfected without mRNA as a control (mock) or with the CSPG4 CAR mRNA (CAR T cells; CAR only). As target cells, either the human TxB cell hybridoma T2.A1 (HLA-A2^+^, CSPG4^−^, gp100^−^) or the human melanoma cell line A375M (HLA-A2^+^, CSPG4^+^, gp100^−^), both either unpulsed or pulsed with the HLA-A2-restricted peptide gp100_280–288_, were used. At 4 h after electroporation, T cells were co-incubated with target cells to assess cytokine production profiles. The specific tumor necrosis factor (TNF) and interferon-gamma (IFNγ) secretion were calculated by subtracting the number of cytokines produced after stimulation with T2.A1, which served as a negative control target cell line (Figure 3). 

Mock-electroporated T cells, which served as a negative control, showed no specific TNF or IFNγ secretion following incubation with any of the abovementioned tumor cells (Figure 3). Stimulation with gp100 peptide-loaded T2.A1 cells (expected TCR response only) revealed significantly higher production of TNF by TCR T cells and TETARs compared to mock-transfected cells, whereas no or only little unspecific cytokine expression was observed in CAR T cells (Figure 3A and Table S2). CAR T cells and TETARs exhibited a significant TNF secretion after co-incubation with unpulsed A375M melanoma cells (expected CAR response only) in comparison to mock-electroporated cells, while no cytokine secretion was observed in TCR T cells (Figure 3A and Table S2). Following stimulation with gp100-pulsed A375M cells (expected TCR and CAR response), CAR T cells, TCR T cells, as well as TETARs showed a significant TNF production when compared to mock-transfected cells (Figure 3A and Table S2). IFNγ secretion patterns were similar to that of TNF (Figure 3B and Table S2). Compared to the T cells transfected with only one receptor, TETARs secreted in general lower quantities of cytokines when stimulated with only one of the two antigens (Figure 3). Of note is, however, that TETARs stimulated with both cognate antigens showed at least the same amount of cytokine secretion (TNF) or a trend towards higher secretion (IFNγ) in comparison to T cells engineered with the CAR only or the TCR alone, indicating an additive effect through recognition of both target antigens by the same T cell (Figure 3). 

Analysis of the anti-inflammatory cytokine interleukin-4 (IL-4) showed only very low production levels with values below 100 pg/mL in all T cell conditions (Figure S2). 

In summary, these results display that TETARs produce significant amounts of pro-inflammatory cytokines after stimulation with target cells expressing either one of the targeted tumor antigens or both.

### 2.4. TETARs Antigen-Specifically Eliminate Tumor Cells

An important characteristic of tumor-specific T cells is their ability to lyse antigen-positive tumor cells. Thus, for cytotoxicity testing, CD8^+^ T cells were lentivirally transduced with the gp100-specific TCR and subsequently electroporated either without mRNA (TCR T cells; TCR only) or with mRNA encoding the CSPG4-specific CAR (TETARs). Non-transduced cells were either mock-transfected (mock) and used as a control or electroporated with the CSPG4 CAR mRNA (CAR T cells; CAR only). Receptor-transfected T cells were then analyzed in a standard ^51^chromium-release assay for their antigen-specific cytotoxicity after incubation with the abovementioned target cells at following effector-to-target cell ratios (E:T): 60:1, 20:1, 6:1, and 2:1. 

**Figure 4:**
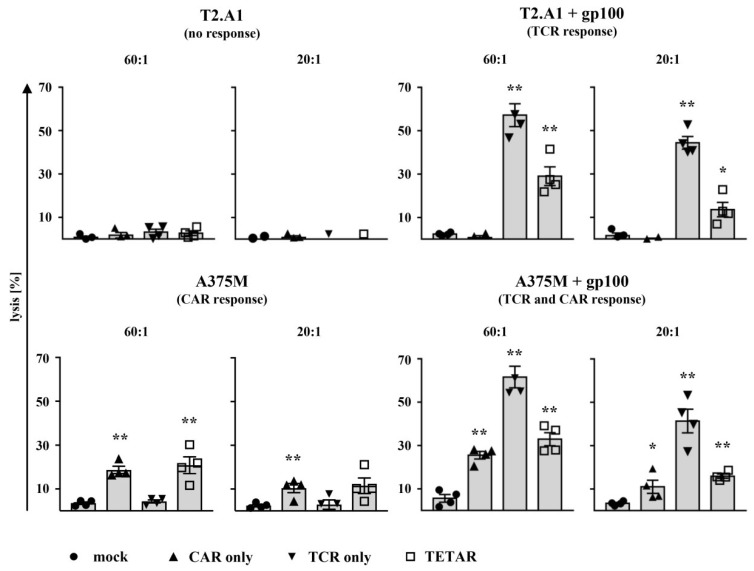
TETARs lyse tumor cells in an antigen-specific manner. CD8^+^ T cells were lentivirally transduced with a gp100-specific TCR (TCR only) and electroporated with mRNA coding for the CSPG4-specific CAR (TETARs), as indicated. Non-transduced T cells were either transfected without mRNA (mock) or with CSPG4-specific CAR mRNA (CAR only). Mock-transfected cells served as a negative control. One day after electroporation, T cells were co-incubated for 4–6 h with the target cell lines T2.A1 (HLA-A2^+^, CSPG4^−^, gp100^−^) and A375M (HLA-A2^+^, CSPG4^+^, gp100^−^), which were either used unpulsed or loaded with gp100 peptide beforehand. Cytotoxicity of T cells was assessed in a ^51^chromium-release assay, and the percentages of lysed cells were determined at the effector-to-target ratios (E:T) of 60:1 and 20:1. Average values of four independent experiments, each additionally depicted as an individual symbol, ± SEM are shown. The *p*-values were calculated by unpaired Student’s *t*-test and are listed in Table S3. ** *p* ≤ 0.01, * *p* ≤ 0.05.

With decreasing effector-to-target cell ratios, a decline in lysis of target cells was observed, as expected (Figure 4 and Figure S3). All T cell conditions showed no or only little unspecific response after incubation with unpulsed T2.A1 cells, which served as negative control target cells (Figure 4 and Figure S3). Stimulation with gp100-loaded T2.A1 cells (expected TCR response only) revealed a significant lysis by TCR T cells and TETARs, while no or only little unspecific background effect was observed in mock or CAR T cells (Figure 4 and Table S3). Lysis of peptide-pulsed T2.A1 cells by TETARs was, however, in general lower when compared to TCR T cells (Figure 4). Following co-incubation with A375M melanoma cells (expected CAR response only), CAR T cells and TETARs exhibited significant cytolytic capacity after antigen encounter at a 60:1 ratio, whereas only few unspecific effects were observed in the case of mock and TCR T cells (Figure 4 and Table S3). In addition, CAR T cells, TCR T cells, and TETARs showed a significantly higher killing of gp100-pulsed A375M melanoma cells (expected TCR and CAR response) at a 60:1 and 20:1 ratio in comparison to mock-electroporated cells, while cytotoxicity of TETARs was in general lower when compared to TCR T cells (Figure 4 and Table S3).

Altogether, these results show that TETARs specifically kill tumor cells expressing either one of the targeted antigens or both through the equipment with a gp100-specific TCR and a CSPG4-specific CAR.

## 3. Discussion

To transfer not only one, but simultaneously two different antigen-specific receptors into the same T cell represents a logical consequence to counteract possible immune escape mechanisms, which may be developed by tumor cells under adoptive T cell therapy. TCRs of CD8^+^ T cells bind to intracellular tumor antigens which upon antigen processing are presented on the cell surface by MHC molecules [17]. In addition, a TCR can also recognize target antigens, which are cross-presented by tumor stromal cells, leading to a more efficient tumor regression [32]. The repertoire of available tumor-specific TCRs has increased in the last decades, as many tumor antigens are expressed intracellularly, and more epitopes have been discovered for the use in adoptive transfer of receptor-transfected cells, especially for the use in solid tumors [33]. CARs consist of an antibody-derived single chain variable fragment (scFv) fused to intracellular domains to provide T cell stimulation [34,35]. A major advantage presented by the use of CARs is their ability to recognize an unprocessed tumor surface antigen independent of MHC restriction and antigen processing [18,19]. The combination of the best of both worlds, the expression of a TCR and a CAR within the same T cell, as well as the combination of stable DNA-based receptor transfer (for receptors which are known to be potent but comparably safe at the same time) and transient RNA-based receptor transfer (for receptors, which are known to be potent, but possibly more dangerous regarding side effects) to generate TETARs, will represent in this context a novel approach in the immunotherapy of cancer, which has yet to be explored.

In our study, it was possible to introduce a gp100-specific TCR into T cells using stable lentiviral transduction, and in addition a second-generation CSPG4-specific CAR into the same T cells by subsequent transient RNA-electroporation. These TETARs were able to secrete cytokines and showed cytotoxicity after stimulation with one of the targeted antigens or both. However, the lytic capacity of TETARs was not as high as that of T cells transfected with the TCR only and unlike in previously reported studies [20,35], no or only little additive or enhanced effect was observed after stimulation with target cells which expressed both target antigens. For further evaluation of a potential benefit of these TETARs, their use in appropriate animal models will be the next important step towards a clinical use in cancer patients.

The approved CD19-directed CAR T cell therapy represents a milestone in cancer immunotherapy for the treatment of therapy-refractory B-cell ALL and diffuse large B-cell lymphoma [6,7]. Patients with a previously poor outcome now have a realistic chance to achieve a complete response and long-term disease remission. However, in a significant number of these patients as well as patients suffering from other types of leukemia and solid tumors, tumor cells are able to develop escape mechanisms under adoptive T cell therapy that lead to resistance and relapse [9,10,11,14,35,36]. For instance, June and colleagues at the University of Pennsylvania observed that up to 60% of B-cell ALL patients may relapse despite CD19-directed CAR T cell persistence [35]. These patients are characterized by the occurrence of CD19-negative leukemia under therapy, most likely due to potent selective pressure by CD19 CAR T cells [35]. Thus, as a potential strategy to counteract the loss of CD19 on leukemia cells, they generated T cells expressing both a CD19-specific CAR and a CD123-specific CAR [35] using stable lentiviral transfection to transfer both receptors into the same T cell. They observed that these engineered T cells prevented antigen loss relapses and revealed higher T cell activation and enhanced anti-tumor efficacy in a mouse model against B-cell ALL compared to T cells expressing CD19 CAR only, CD123 CAR only, or a pooled combination of both CD19 or CD123 CAR T cell populations [35]. In another study, Slaney et al. introduced two receptors, a Her2/ERBB2-specific CAR and additionally a gp100-specific TCR, into the same T cell [37]. They used a regimen of adoptive cell transfer incorporating vaccination (ACTIV) with recombinant vaccinia virus expressing gp100 to treat a range of tumors including breast tumors and large liver tumors and observed a massive infiltration of T cells into the tumor, resulting in durable complete remission of Her2^+^ tumors in mice [37].

However, a drawback of CAR T cell therapy is the presence of on-target off-tumor toxicities, depending on the used target antigen [24]. In the approved CD19 CAR T cell therapy, loss of healthy CD19^+^ cells can be compensated by supplementing intravenous immunoglobulins. The CD123 antigen, for instance, which was used in the above discussed study, is known to be also expressed on non-malignant cells [24]. Thus, although anti-CD123 CAR T cell therapy proved to be efficient for the treatment of acute myeloid leukemia (AML) in a preclinical setting [38,39], its clinical use is impeded due to CD123 expression on healthy stem cells [40]. Furthermore, Her2/ERBB2, which was used by Slaney et al. as the target antigen of their CAR construct [37], is known to be expressed on lung epithelial cells, which might cause fatal side effects [24]. For instance, a patient with colon cancer metastatic to the lungs and liver received Her2/ERBB2-specific CAR T cells [41] and within 15 minutes after cell infusion the patient experienced respiratory distress and displayed a dramatic pulmonary infiltrate on a chest X-ray [41]. Despite intensive medical intervention, the patient died 5 days after treatment [41]. The administered cells most likely localized to the lungs immediately following infusion and were triggered to release cytokines by the recognition of low levels of ERBB2 on lung epithelial cells, which then caused the fatal side effects [41].

A possible strategy to bypass these hurdles and still use the dual-CAR or TCR+CAR expressing T cells by Ruella et al. and Slaney et al. for further clinical development could be the combined DNA- and RNA-based receptor transfer [35,37]. In this context the “safer” receptor, which is lentivirally transduced into the T cells using receptor-encoding DNA (e.g., the CD19 CAR or the gp100 TCR) should have a strong and permanent anti-tumor effect, whereas the “more dangerous” receptor, that is transfected into the same T cell using receptor-encoding RNA (e.g., the CD123 CAR or Her2/ERBB2 CAR), should have an additional “boost” effect at the beginning of the therapy. This could massively increase the pressure on the tumor by enhanced induction of direct tumor-cell killing and rapid on-site T cell expansion and might be an effective and fast way to eradicate large quantities of tumor cells before tumor cells might escape immune recognition.

## 4. Materials and Methods

### 4.1. Cells

Blood was collected from healthy donors after informed consent and approval by the institutional review board of the Friedrich-Alexander-University (FAU) of Erlangen-Nürnberg (Reference Number: 65_16 B) had been obtained. First, peripheral blood mononuclear cells (PBMCs) were extracted using density centrifugation via Lymphoprep reagent (Axis-Shield, Oslo, Norway). CD8^+^ T cells were then obtained via MACS according to manufacturer’s instructions (Miltenyi, Bergisch-Gladbach, Germany). Purified T cells were cultured in X-Vivo 15 medium already containing L-glutamine, gentamycin, and phenol red (Biozym Scientific GmbH, Hessisch Oldendorf, Germany). Target cell lines included the TxB cell hybridoma T2.A1 (HLA-A2^+^, CSPG4^−^, gp100^−^ [20]; kind gift from Prof. Dr. Schulz, Nuremberg, Germany) and the melanoma cell line A375M (HLA-A2^+^, CSPG4^+^, gp100^−^ [20]; kind gift from Dr. Aarnoudse, Leiden, Netherlands; ATCC CRL-3223). Prior to co-incubation with T cells, we cultured the target cells in R10 medium (RPMI 1640 (Lonza, Basel, Switzerland) supplemented with 2 mM L-glutamine (Lonza), 100 IU/mL penicillin (Lonza), 100 mg/ml streptomycin (Lonza), 10% (v/v) heat-inactivated fetal calf serum (PAA, GE healthcare, Piscataway, NY, USA), 2 mM HEPES (PAA, GE healthcare, Little Chalfont, UK), and 2 mM β-mercaptoethanol (Gibco, Life Technologies, Carlsbad, CA, USA)). The above-mentioned target cells were additionally pulsed with HLA-A2-restricted peptide gp100_280–288_ (YLEPGPVTA) as previously described [16], where indicated. Peptide-loading was performed in DC-medium (RPMI 1640 (Lonza) supplemented with 1% heat-inactivated human serum (Sigma-Aldrich, Taufkirchen, Germany), 2 mM L-glutamine (Lonza), and 0.04% of 20 mg/L gentamycin (Lonza)). 

### 4.2. Lentiviral Transduction of T Cells

The gp100 TCR α- and β-chains were encoded in a lentiviral vector (pcLV-EF1a-MCS-WPRE) and expressed under control of an EF1a promotor (designed by and purchased from Sirion Biotech, Planegg-Martinsried, Germany). Lentiviral transduction of T cells was performed as follows: After MACS-isolation, T cells were subsequently stimulated with 0.1 µg/mL anti-CD3 antibody OKT-3 (Orthoclone OKT-3; Janssen-Cilag, Neuss, Germany), 0.25 µg/mL anti-CD28 antibody (BD Biosciences, Franklin Lakes, NJ, USA), and 1000 IU/mL interleukin-2 (IL-2) (Proleukin; Novartis, Nuremberg, Germany). Two days later, T cells were lentivirally transduced with a gp100-specific TCR using a multiplicity of infection (MOI) of 10 and the transduction enhancer LentiBOOST^TM^ (consisting of 1000 µg/mL P338 and 10 µg/mL polybrene, Sirion Biotech, Planegg-Martinsried, Germany) together with 1000 IU/mL IL-2 (Novartis) via spinoculation (800 g for 90 min). As a negative control, only IL-2 was added to the cells prior to spinoculation. On the following day, culture medium of T cells was replaced by fresh X-Vivo 15 medium, and 5 ng/mL IL-7 (PeproTech, Rocky Hill, NJ, USA), 5 ng/mL IL-15 (Miltenyi), and 1000 IU/mL IL-2 (Novartis) were added. After three days, T cells were antigen-specifically stimulated with irradiated (140 gray for 6 min) and gp100 peptide-pulsed A375M target cells for one week and subsequently stimulated for another week. Non-transduced cells were re-stimulated with 0.1 µg/mL OKT-3 (Janssen-Cilag), 0.25 µg/mL anti-CD28 antibody (BD Bisosciences, Franklin Lakes, NJ, USA), and 1000 IU/mL (Novartis). New culture medium and IL-2 was added to the cells at day four after each stimulation.

### 4.3. RNA Production and Transfection

The mMESSAGE mMACHINE T7 Ultra Transcription Kit (Life Technologies, Carlsbad, CA, USA) was used for the generation of mRNA. The mRNA was further purified with the RNeasy Kit (Qiagen, Hilden, Germany) according to manufacturer’s instructions. Then, T cells were electroporated with the generated mRNA encoding a CSPG4-specific CAR (MCSP_HL_ CD28-CD3ζ) [42] by the GenePulser Xcell system (Bio-Rad, Hercules, CA, USA) with the square-wave protocol and 500 V for 5 ms, as previously described [43]. Following electroporation, cells were rapidly transferred to X-Vivo 15 medium.

### 4.4. Receptor Expression Analysis of Engineered T Cells

The goat-F(ab’)2 anti-human IgG antibody (Southern Biotech, Birmingham, AL, USA) directed against the extracellular IgG1 CH2-CH3 CAR domain was used to determine the CAR expression on T cells. TCR expression on the cell surface of T cells was analyzed using an MHC Dextramer (HLA-A*0201/YLEPGPVTV; Immudex, Copenhagen, Denmark) directed against the gp100-specific TCR. In addition, the anti-7-AAD antibody (BD Biosciences) was used to exclude nonviable T cells. The detailed procedure of cell surface staining was previously described [44]. Immunofluorescence was measured via the FACS Calibur (BD Biosciences, Heidelberg, Germany), which was equipped with the CellQuest Pro software (BD Biosciences). Data were analyzed using the FCS Express software, version 5 (DeNovo Software, Glendale, CA, USA).

### 4.5. Cytokine Secretion Analysis of Engineered T Cells

Cytokine production of T cells was analyzed as previously described [45]. In brief, transfected T cells were co-incubated with target cell lines T2.A1 and A375M (either unpulsed or pulsed with the HLA-A2-restricted peptide gp100_280–288_) overnight at a 1:1 effector-to-target cell ratio. Cytokine concentrations of TNF, IFNγ, and IL-4 in the supernatants were measured utilizing the Th1/Th2 Cytometric Bead Array Kit II (BD Biosciences) according to manufacturer’s instructions. Immunofluorescence was detected using the FACSCanto II (BD Biosciences) equipped with FACSDiva software (BD Biosciences). Data were analyzed via FCS Express software, version 5 (DeNovo Software).

### 4.6. Cytotoxicity Analysis of Engineered T Cells

Cytolytic capacity of transfected T cells was assessed with a standard 4–6 h ^51^chromium-release assay, as previously described [16]. First, target cell lines T2.A1 and A375M were labelled with 20 µCi of Na_2_^51^CrO_4_/10^6^ cells (Perkin Elmer, Waltham, MA, USA) for one hour. Subsequently, half of the target cells were pulsed with the HLA-A2-restricted peptide gp100_280–288_ for one hour. Transfected T cells and target cells were co-incubated to obtain the following effector-to-target cell ratios (E:T): 60:1, 20:1, 6:1, and 2:1. Chromium release in supernatants was analyzed with the Wallac 1450 MicroBeta plus Scintillation Counter (Wallac, Turku, Finland). The percentage of lysis was calculated as follows: [(measured release – background release)/(maximum release − background release)] × 100%.

### 4.7. Figure Preparation and Statistical Analysis

Graphs were created and statistical analysis was performed using GraphPad Prism, version 7 (GraphPad Software, La Jolla, CA, USA). The *p*-values were analyzed using the unpaired Student’s *t*-test, assuming a Gaussian distribution. * indicates *p* ≤ 0.05 and ** indicates *p* ≤ 0.01.

## 5. Conclusions

We have shown here that it is feasible to co-transfect the same T cells with a TCR specific for gp100 and a CAR specific for CSPG4 using a combined DNA- and RNA-based receptor transfer to generate TETARs for the use in adoptive T cell therapy of cancer. These TETARs proved to be functional regarding cytokine secretion and cytolytic activity upon stimulation with each of their cognate antigens. The confirmation that two antigen-specific receptors can be functionally combined using two different methods to introduce each receptor into the same T cell may open up new possibilities and opportunities in cancer immunotherapy, which should be further evaluated in suitable preclinical models towards a potential use in cancer patients.

## Supplementary Materials

The following are available online at https://www.mdpi.com/2072-6694/11/5/696/s1, Figure S1: Gp100 TCR and CSPG4 CAR expression of TETARs, Figure S2: Antigen-specific IL-4 production of TETARs, Figure S3: TETARs show antigen-specific cytotoxicity. Table S1: *p*-values corresponding to Figure 2C, Table S2: *p*-values corresponding to Figure 3, Table S3: *p*-values corresponding to Figure 4 and Figure S3.
