# Supplementary material for: Arming T Cells with a gp100-Specific TCR and a CSPG4-Specific CAR Using Combined DNA- and RNA-Based Receptor Transfer
Source: Cancers (Basel). 2019 May 20;11(5):696. doi: 10.3390/cancers11050696 (PMC6562862; doi:10.3390/cancers11050696)
Supplement: Supplementary file 1 [file cancers-11-00696-s001.pdf]

Supplementary Materials: Arming T Cells with a gp100-Specific TCR and a CSPG4-Specific CAR Using Combined DNA- and RNA-Based Receptor Transfer

Bianca Simon, Dennis C. Harrer, Beatrice Schuler-Thurner, Gerold Schuler and Ugur Uslu

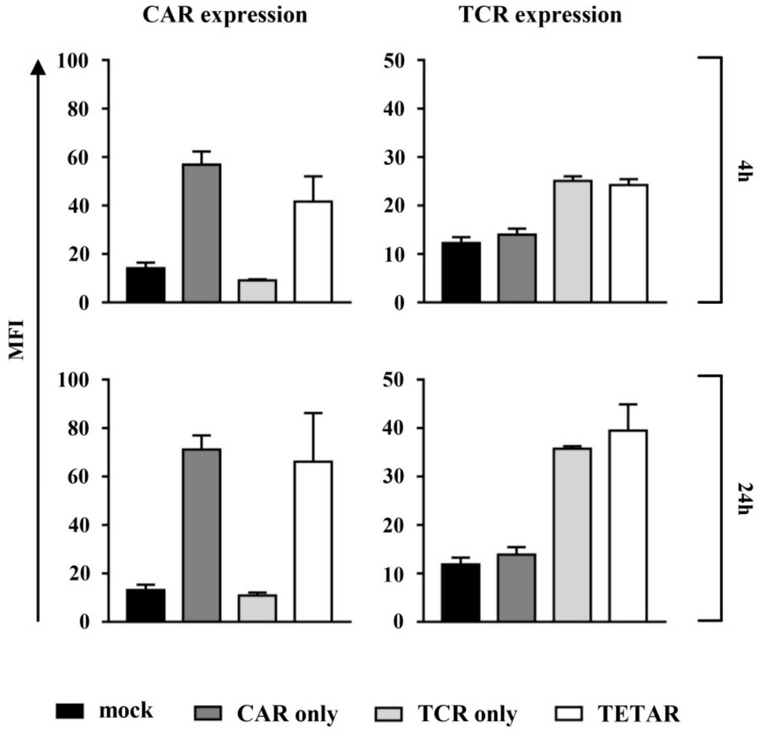

Figure S1. Gp100 TCR and CSPG4 CAR expression of TETARs. CD8+ T cells were lentivirally transduced with a gp100-specific TCR (TCR only) and electroporated with mRNA coding for the CSPG4-specific CAR (TETARs), as indicated. Non-transduced T cells were either transfected without mRNA (mock) or with CSPG4-specific CAR mRNA (CAR only). Mock-transfected cells served as negative control. The surface expression of the gp100-specific TCR and the CSPG4-specific CAR were assessed in a time-course experiment. Receptor expression levels were measured at 4 and 24 hours after electroporation. Average geometric mean values of CAR and TCR stainings of 3 independent experiments with SEM are shown.

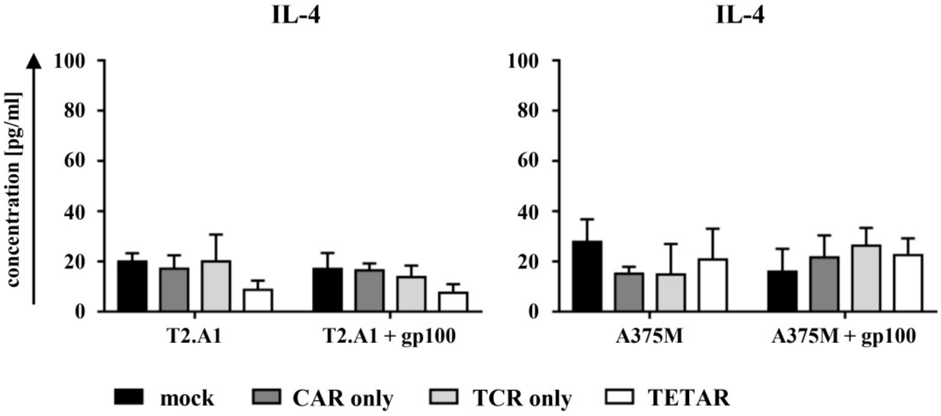

Figure S2. Antigen-specific IL-4 production of TETARs. CD8+ T cells were lentivirally transduced with a gp100-specific TCR (TCR only) and electroporated with mRNA coding for the CSPG4-specific CAR (TETARs). Non-transduced T cells were either transfected without mRNA (mock) or with CSPG4-specific CAR mRNA (CAR only). Mock-transfected cells were used as negative control. T cells were co-incubated overnight with either gp100 peptide-loaded or unpulsed T2.A1 (HLA-A2+, CSPG4−, gp100−)

and A375M (HLA-A2$^+$, CSPG4$^+$, gp100$^-$) target cells. The production of IL-4 was measured in a cytometric bead array (CBA). Mean values of 4 independent experiments with SEM are shown.

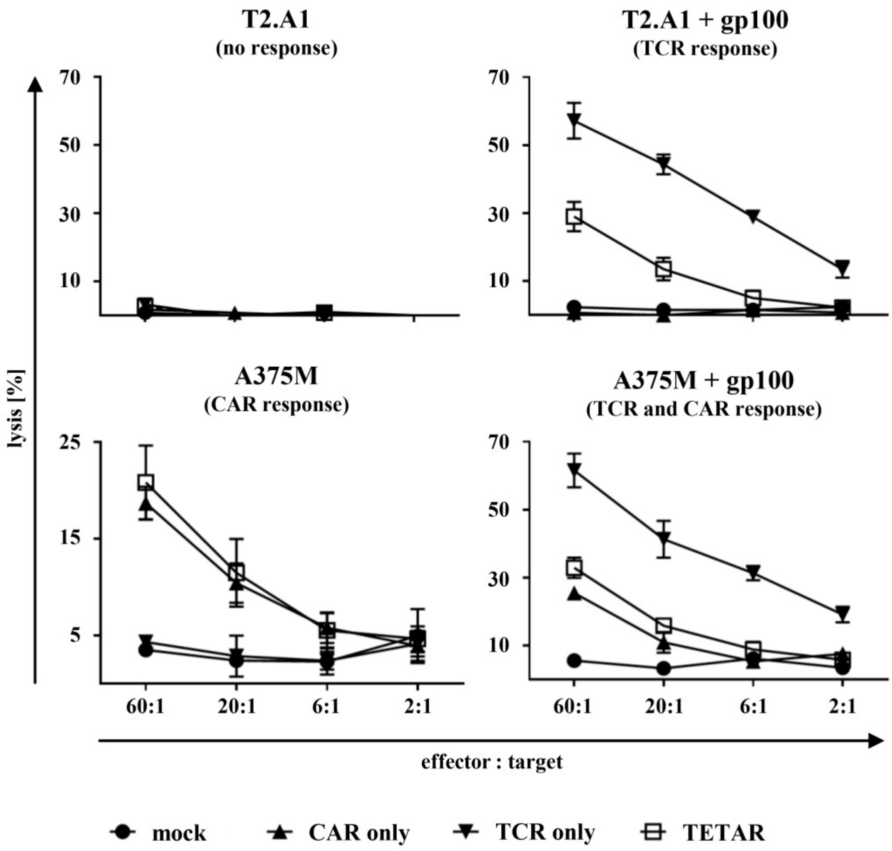

Figure S3. TETARs show antigen-specific cytotoxicity**.** CD8$^+$ T cells were lentivirally transduced with a gp100-specific TCR (TCR only) and electroporated with mRNA coding for the CSPG4-specific CAR (TETARs). Non-transduced T cells were either transfected without mRNA (mock) or with CSPG4-specific CAR mRNA (CAR only). Mock-transfected cells were used as negative control. One day after electroporation, T cells were co-incubated for 4-6 hours with either gp100 peptide-loaded or unpulsed T2.A1 (HLA-A2$^+$, CSPG4$^-$, gp100$^-$) and A375M (HLA-A2$^+$, CSPG4$^+$, gp100$^-$) target cells. Lytic capacity of T cells was examined in a ^{51}chromium-release assay and the percentages of lysed cells were calculated at following effector to target ratios (E:T): 60:1, 20:1, 6:1, 2:1. Mean values of 4 independent experiments ± SEM are shown. The *p*-values were calculated by unpaired Student's *t*-test and are listed in Table S3.

Table S1. *p*-values [1] corresponding to Figure 2C.

**Conditions CAR$^+$**	**Time points**		
	**4h**	**8h**	**24h**
CAR only vs. TETAR	ns	ns	ns

[1] calculated by unpaired Student's *t*-test from 3 independent experiments, ns $p > 0.1$.

Table S2. *p*-values [1] corresponding to Figure 3.

**Conditions TNF**	**T2.A1 + gp100**	**A375M**	**A375M + gp100**
mock vs CAR only	ns	**	**
mock vs TCR only	*	ns	**
mock vs TETAR	*	*	*
Conditions IFNγ	T2.A1 + gp100	A375M	A375M + gp100
mock vs CAR only	ns	0.0799	0.0904
mock vs TCR only	**	ns	**
mock vs TETAR	ns	ns	0.0736

[1] calculated by unpaired Student's *t*-test from 4 independent experiments, ** $p \leq 0.01$, * $p \leq 0.05$, ns $p > 0.1$, p-values between 0.05 and 0.1 are specified.

Table S3. p-values [1] corresponding to Figure 4 and S3.

**Conditions 60:1**	**T2.A1**	**T2.A1 + gp100**	**A375M**	**A375M + gp100**
mock vs CAR only	ns	ns	**	**
mock vs TCR only	ns	**	ns	**
mock vs TETAR	ns	**	**	**
Conditions 20:1	T2.A1	T2.A1 + gp100	A375M	A375M + gp100
mock vs CAR only	ns	ns	**	*
mock vs TCR only	ns	**	ns	**
mock vs TETAR	ns	*	0.0429	**
Conditions 6:1	T2.A1	T2.A1 + gp100	A375M	A375M + gp100
mock vs CAR only	ns	ns	ns	ns
mock vs TCR only	ns	**	ns	**
mock vs TETAR	ns	ns	ns	ns
Conditions 2:1	T2.A1	T2.A1 + gp100	A375M	A375M + gp100
mock vs CAR only	ns	ns	ns	*
mock vs TCR only	ns	**	ns	**
mock vs TETAR	ns	ns	ns	ns

[1] calculated by unpaired Student's *t*-test from 4 independent experiments, ** $p \leq 0.01$, * $p \leq 0.05$, ns $p > 0.1$, p-values between 0.05 and 0.1 are specified.

© 2019 by the authors. Licensee MDPI, Basel, Switzerland. This article is an open access article distributed under the terms and conditions of the Creative Commons Attribution (CC BY) license (http://creativecommons.org/licenses/by/4.0/).
